# Palmitoyl ascorbic acid 2-glucoside has the potential to protect mammalian cells from high-LET carbon-ion radiation

**DOI:** 10.1038/s41598-018-31747-1

**Published:** 2018-09-14

**Authors:** Alexis H. Haskins, Dylan J. Buglewicz, Hirokazu Hirakawa, Akira Fujimori, Yasushi Aizawa, Takamitsu A. Kato

**Affiliations:** 1 Department of Environmental & Radiological Health Sciences, Colorado State University, 1618 Campus Delivery, Fort Collins, CO 80523 USA; 2 National Institute of Radiological Sciences, National Institutes for Quantum and Radiological Science and Technology, Chiba, 263-8555 Japan; 3 Department of Planning & Development, Carlit Holdings Co. Ltd., 1-17-10 Kyobashi, Chuo-ku, Tokyo, 104-0031 Japan

## Abstract

DMSO, glycerol, and ascorbic acid (AA) are used in pharmaceuticals and known to display radioprotective effects. The present study investigates radioprotective properties of novel glyceryl glucoside, ascorbic acid 2-glucoside, glyceryl ascorbate, and palmitoyl ascorbic acid 2-glucoside (PA). Gamma-rays or high-LET carbon-ions were irradiated in the presence of tested chemicals. Lambda DNA damage, cell survival, and micronuclei formation of CHO cells were analyzed to evaluate radioprotective properties. Radiation-induced Lambda DNA damage was reduced with chemical pre-treatment in a concentration-dependent manner. This confirmed tested chemicals were radical scavengers. For gamma-irradiation, enhanced cell survival and reduction of micronuclei formation were observed for all chemicals. For carbon-ion irradiation, DMSO, glycerol, and PA displayed radioprotection for cell survival. Based on cell survival curves, protection levels by PA were confirmed and comparable between gamma-rays and high-LET carbon-ions. Micronuclei formation was only decreased with AA and a high concentration of glycerol treatment, and not decreased with PA treatment. This suggests that mechanisms of protection against high-LET carbon-ions by PA can differ from normal radical scavenging effects that protect DNA from damage.

## Introduction

Ionizing radiation (IR) of living cells is a known cause of cancer, mutations, and aging^1–4^. IR can produce highly reactive free radicals. Most free radicals that damage cellular systems are reactive oxygen species (ROS)^1–4^. Free radicals disrupt cell’s DNA and produce single and double strand DNA breaks, which result in chromosome aberrations and cell death^1,3,4^. IR effects can be reduced by the pre-treatment of antioxidants. Antioxidants can neutralize oxidative stress by scavenging free radicals prior to damaging DNA^2^. Dimethyl sulfide (DMSO), glycerol, and ascorbic acid (AA) are well known radical scavengers^5–10^. However, they display cellular toxicity with continuous exposure^11–13^. Less toxic radioprotectors may be useful to reduce the damaging effects of IR on healthy tissues surrounding tumors during radiotherapy. Moreover, practical radioprotectors may also be useful to protect radiation workers who are exposed to low levels of IR periodically.

Recently, glycosyl and glyceryl modified chemicals are available for glycerol and AA. They are stable in water and showed less cytotoxicity. In our previous study, glycosylation has a positive effect for flavonoids radioprotection *in vitro*^14^. Glyceryl glucoside (GG) is a glycosyl derivative of glycerol and glucose^15,16^. GG exhibits antioxidant properties by decreasing ROS and is associated with cell regeneration and renewal^17^. GG is naturally present in traditional Japanese foods brewed using koji (i.e. sake and miso), wine, and cosmetics (i.e. anti-aging and skin moisturizing) as an osmoprotectant^16–19^. Our previous study displayed GG has lower cytotoxicity compared to DMSO and glycerol^16^. A recent toxicology study reported that *in vitro* and *in vivo*, GG exhibited non-mutagenic and non-cytotoxic properties^17^. Glycosylated and glycerylated AA, which are used in food and supplements as vitamins and antioxidants^20^ are: ascorbic acid 2-glucoside (2G); glycerol ascorbic acid (GA); and palmitoyl ascorbic acid-2-glucoside (PA). Previous studies showed AA modified chemicals maintained their antioxidant properties and are expected to be potential practical radioprotectors^9,21,22^.

**Figure 1:**
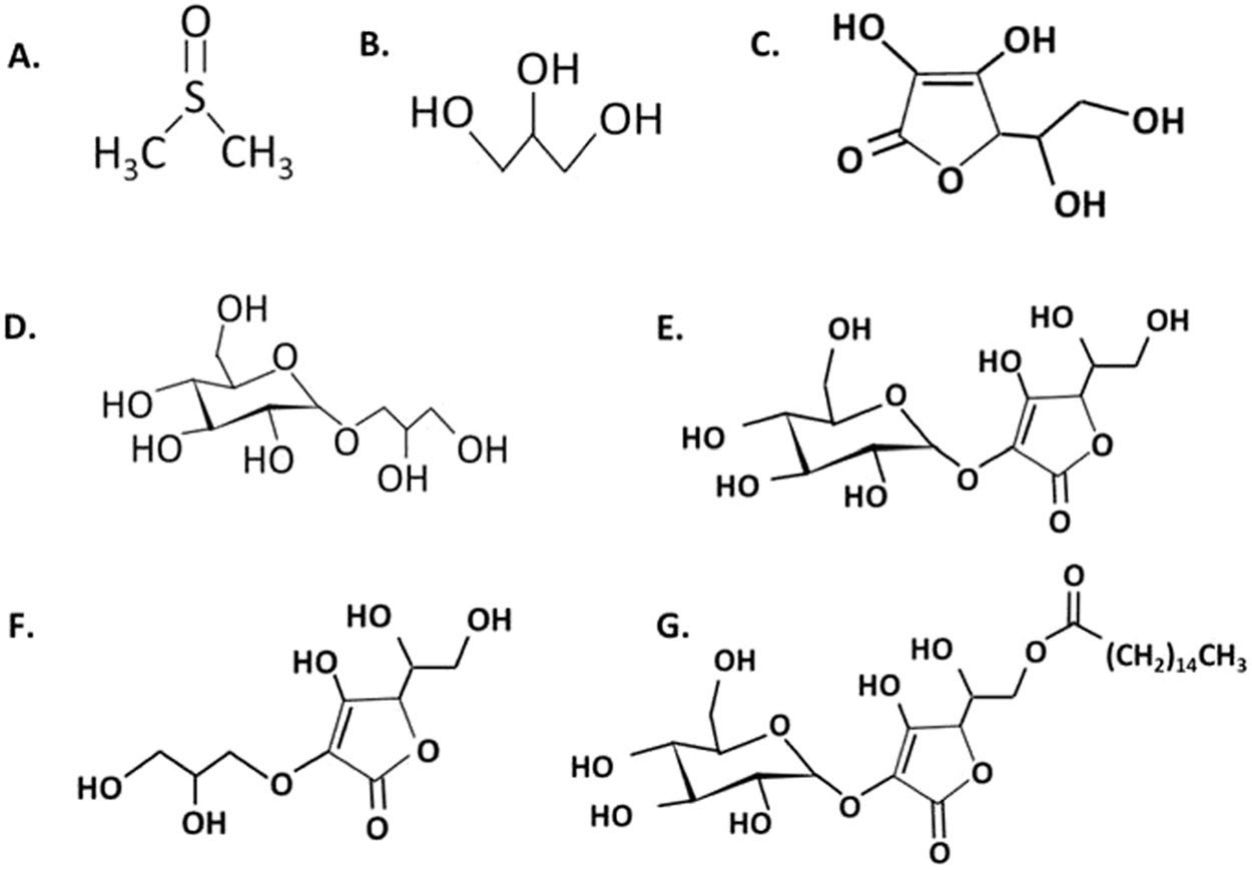
Chemical structures of (**A**) dimethyl sulfoxide, (**B**) glycerol, (**C**) ascorbic acid, (**D**) glyceryl glucoside, (**E**) ascorbic acid 2-glucoside, (**F**) glyceryl ascorbate, and (**G**) palmitoyl ascorbic acid 2-glucoside.

In this study, we hypothesized that chemically modified classical radioprotectors are also radical scavengers and have the potential to display radioprotective effects. The current study explored the radioprotective properties of DMSO, glycerol, GG, AA, 2 G, GA, and PA (Fig. 1) by investigating CHO cells and Lambda DNA in terms of cell survival, micronuclei formation, and DNA damage after irradiation of gamma-rays and high-LET carbon-ions.

## Results

### *In vitro* radioprotective ability of tested chemicals

**Figure 2:**
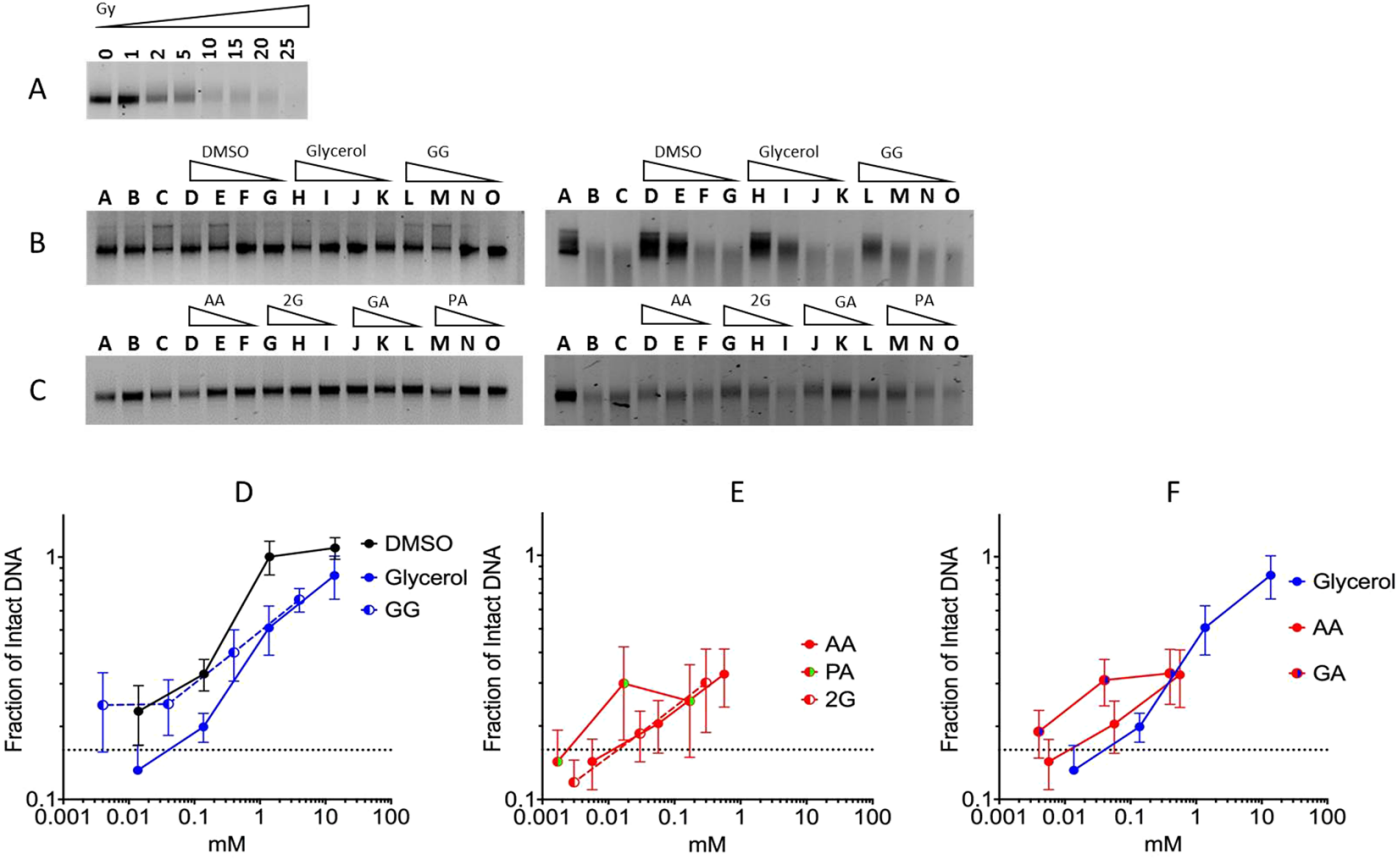
Gel electrophoresis of Lambda DNA. (**A**) Radiation dose-dependent DNA damage. (**B** left, and **C** left) Effects of tested chemicals on DNA without irradiation. (**B** right) Radioprotective effects of pre-treated DMSO, glycerol, and GG. Triangles indicate concentration gradient. Lane A: control water without irradiation; lane B and C water with irradiation; lane D–G DMSO at 14 mM, 1.4 mM, 0.14 mM, and 0.014 mM; lane H-K: glycerol at 13.7 mM, 1.37 mM, 0.137 mM, and 0.0137 mM; lane L–O: GG at 4 mM, 0.4 mM, 0.04 mM, and 0.004 mM. (**C** right) Radioprotective effects of AA, 2G, GA, and PA. Triangles indicate concentration gradient. Lane A: control water without irradiation; lane B and C: water with irradiation; lane D–F AA at 0.56 mM, 0.056 mM, and 0.0056 mM; lane G–I: 2G at 0.3 mM, 0.03 mM, and 0.003 mM; lane J–L: GA at 0.4 mM, 0.04 mM, and 0.004 mM; lane M–O: PA at 0.17 mM, 0.017 mM, and 0.0017 mM. **(D–G)** Fraction of intact irradiated Lambda DNA with chemicals. Error bars indicate standard error of the means. At least three independent experiments were carried out. The cropped gel images were used in the figure, and full-length gel images are presented in Supplementary Fig. S1.

In the gel electrophoresis assay, irradiated and fragmented Lambda DNA migrated further and was detected as a smear in a dose dependent manner (Fig. 2A). In Fig. 2A, Lambda DNA was damaged at 20 Gy. We confirmed the tested chemicals, alone, did not produce DNA damage (Fig. 2B left, and C left). Chemical dose-dependent protection was observed as a reduction of DNA damage (Fig. 2B right, and C right). All agents displayed positive radioprotective abilities at 0.1 mM concentrations compared to the control (Fig. 2D–F). DMSO displayed the highest radioprotective properties at 1.4 and 14 mM. Glycerol and GG had nearly identical protection at 1 mM and above (Fig. 2D). AA and 2 G exhibited similar protective properties (Fig. 2E), as well as GA and PA (Fig. 2E,F). Gel electrophoresis results confirm the tested chemicals are radical scavengers.

### Radioprotection of cell survival

**Figure 3:**
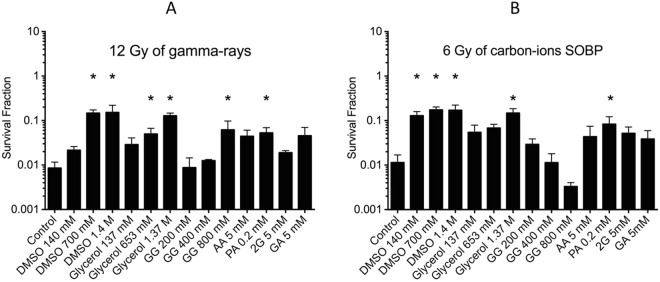
DMSO, glycerol, GG, AA and its derivatives on radioprotective effects for cell survival of CHO cells. (**A**) Under 12 Gy gamma-ray irradiation, cells irradiated without chemicals induced a 0.0086 cell survival fraction. (**B**) Under 6 Gy high-LET carbon-ion irradiation, cells irradiated without chemicals stimulated a 0.018 cell survival rate. * indicate statistical significance (P < 0.05). Error bars indicate standard error of the means. At least three independent experiments were carried out.

To evaluate the role of DMSO, glycerol, GG, AA and its derivatives as potential radioprotectors, cell survival was investigated using colony formation assay. CHO cells were treated with various non-toxic chemical concentrations for 0.5 h and then exposed to 12 Gy gamma-rays or 6 Gy high-LET carbon-ions. For gamma-rays (12 Gy), without chemicals cell survival fraction was approximately 0.0086 (Fig. 3A). In Fig. 3A, cells were pre-treated with various chemicals and then irradiated with 12 Gy gamma-rays; all displayed an overall increased cell survival compared to the control. CHO cells pre-treated with DMSO and glycerol presented radioprotection, significantly increasing cell survival above 100 mM (P < 0.05). However, GG required a concentration greater than 400 mM to show similar protection (P = 0.0195). 5 mM of AA and its derivatives (2G, GA) and 0.2 mM PA demonstrated radioprotection. Especially PA showed significant protection. For high-LET carbon-ion radiation (6 Gy), without chemicals cell survival fraction was approximately 0.018 (Fig. 3B). Cells pre-treated with greater than 140 mM DMSO and 1.37 M glycerol, then irradiated with 6 Gy high-LET carbon-ions, revealed significant radioprotection (P = 0.00327 for 140 mM DMSO, P = 0.0033 for 1.37 M glycerol). GG, AA, 2G, PA and GA did not show significant radioprotection in the tested concentrations. 0.2 mM PA showed increased cell survival, but it was not statistically significant (P = 0.3324).

### Micronuclei formation

**Figure 4:**
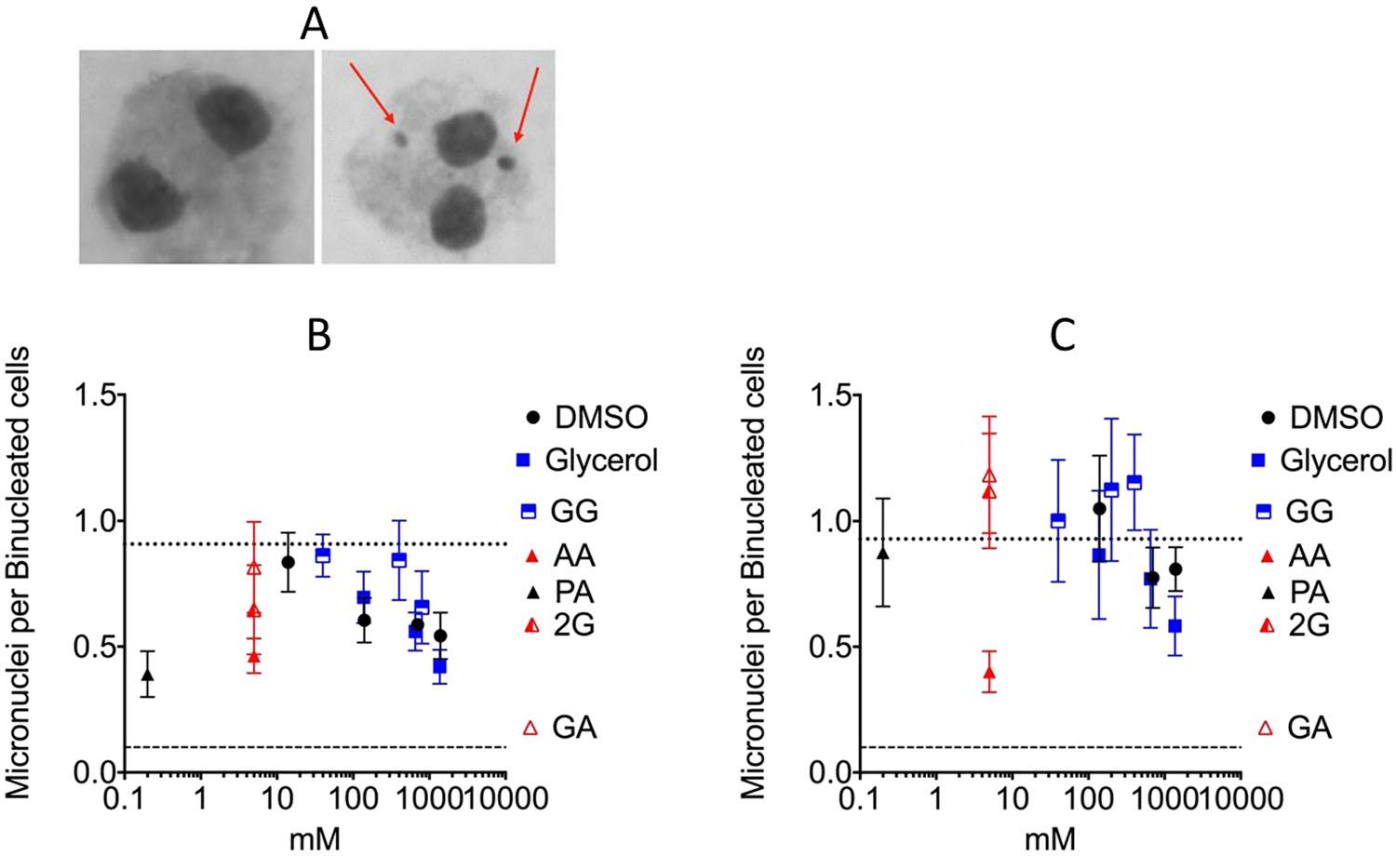
Radioprotective effects of DMSO, glycerol, GG, AA, 2G, GA, and PA by micronuclei assay. (**A**) On the left is a normal binucleated cell. On the right is binucleated cell with micronuclei formation indicated by red arrows. (**B**) Micronuclei formation per binucleated CHO cell with pre-treated chemicals after gamma-ray irradiation. Horizontal dashed line indicates non-irradiated control (less than 0.1 micronuclei per binucleated cells for each condition). Horizontal dotted line indicates the control (0.91 micronuclei per binucleated cells). (**C**) Micronuclei formation after high-LET carbon-ion irradiation. Horizontal dashed line indicates non-irradiated control (less than 0.1 micronuclei per binucleated cells for each condition). Horizontal dotted line indicates the control (0.93 micronuclei per binucleated cells). Error bars indicate standard error of the means. At least three independent experiments were carried out.

To investigation the role of DMSO, glycerol, GG, AA and its derivatives, in the prevention of radiation-induced genotoxicity, micronuclei formation was analyzed. For gamma-ray irradiation (6 Gy), without chemical treatment CHO cells show a level of 0.91 micronuclei per binucleated cell (Fig. 4B). All pre-treated chemicals demonstrated radioprotection against micronuclei formation compared to the control. 1.4 mM DMSO and 1.37 mM glycerol displayed a strong reduction of radiation-induced micronuclei formation (P = 0.0263, P = 0.037 respectively). 5 mM of AA and 0.2 mM of PA reduced micronuclei formation over 50% compared to the control. The most significant reduction in micronuclei formation was displayed by 0.2 mM of PA (P = 0.0142), with 0.26 micronuclei per binucleated cell. For high-LET carbon-ion radiation (3 Gy), without chemical treatment CHO cells produced 0.93 micronuclei per binucleated cell (Fig. 4C). At varying concentrations, all chemicals did not reduce micronuclei formation compared to the control, with the exception of AA (5 mM) and glycerol (1.37 mM). AA (5 mM) reduced micronuclei formation by 50% with 0.42 micronuclei per binucleated cell, but it was not significant (P = 0.2294).

### Survival curve for palmitoyl ascorbic acid-2-glucoside

**Figure 5:**
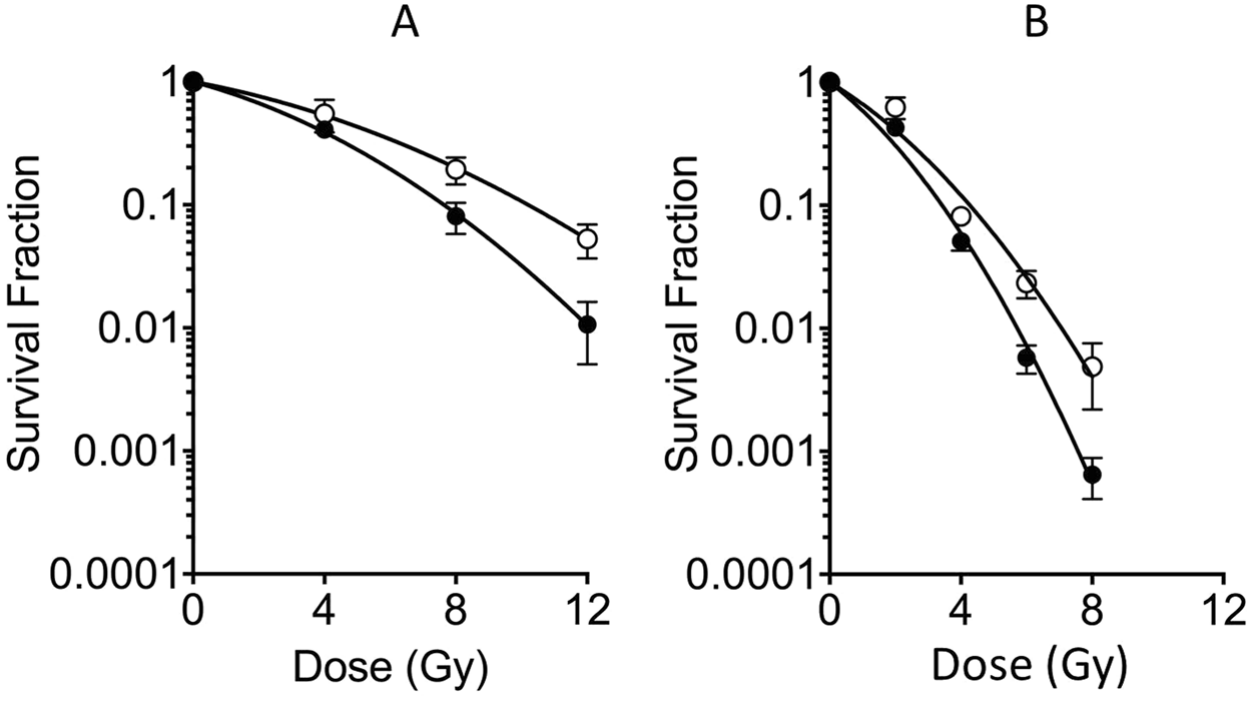
Cell survival curves with and without 0.2 mM palmitoyl ascorbic acid 2-glucoside (PA). (**A**) gamma-ray irradiation. (**B**) Carbon-ions irradiation. Closed circles indicate control; open circles indicate PA. Curves were fitted to liner-quadratic model. Error bars indicate standard error of the means. At least three independent experiments were carried out.

In order to confirm the radioprotective properties of PA, survival curves with and without 0.2 mM PA treatment were created. PA showed radioprotective properties for both gamma-rays and high-LET carbon-ions SOBP (Spread out Bragg peak) irradiation (Fig. 5A,B). D_10_ values were increased from 7.64 Gy to 9.64 Gy for gamma-ray irradiation and 3.43 Gy to 4.27 Gy for high-LET carbon-ion irradiation. The PER was 1.26 for gamma-rays and 1.24 for high-LET carbon-ion irradiation. Therefore, the level of protection by PA was similar between gamma-rays and high-LET carbon-ions.

## Discussion

As expected, DMSO, glycerol and AA exhibited radioprotection under gamma-ray (low-LET) and carbon-ion (high-LET) irradiation. GG demonstrated radical scavenging effects *in vitro* at 4 mM solution, but not for cell survival. This may be due to glycosylation preventing entry into the cell membrane *in vivo*, since glycosylated chemicals are more water soluble, making them less bioavailable^23,24^. GG is glycosylated; thus, we suggest deglycosylation must occur prior or during cell-chemical uptake in order for α-glucosidase to initiate digestion, increasing cellular uptake *in vivo*^24–26^. In this study, cells were pre-treated 0.5 hour prior to irradiation, which was sufficient for DMSO, glycerol, and AA to display radioprotective properties. It is possible that GG requires a longer pre-treatment to efficiently be bioavailable. Perhaps, a greater concentration of GG is needed to be radioprotective for the current treatment time, although concentrations of GG greater than 800 mM display cytotoxic effects^14^. Thus, GG does not display comparable radioprotection like DMSO or glycerol in non-toxic concentrations. It is important to note that the concentration of each chemical largely relied on *in vitro* DNA damage versus cell culture experiments. *In vitro* DNA experiments required more dilute concentrations than cell culture experiments, due to the ease that chemicals have when entering DNA compared to the cell membrane (Figs 2 and 4).

Furthermore, 2G did not display strong radioprotection (Fig. 4B,C), as well as AA because glucoside inhibits cell entry. We expected enhanced protection by GA because both AA and glycerol presented radioprotection. However, GA exhibited protection only for gamma-rays (Fig. 4A) and displayed better protection for DNA damage (Fig. 2D), as it is a glycerylated AA. Cell survival for GA was not increased as expected since GA may not have been up-taken by the cell. This may be due to glycerol modification of AA not enhancing bioavailability^14^. On the other hand, PA is a great radioprotector (Figs 2E, 3A,B and 4B,C) even with glycosyl substituent as 2G. PA is a liposoluble ester of ascorbic acid^20^, and we confirmed PA displays protection at all doses (Fig. 5). It is possible that liposoluble properties enhanced PA’s cellular uptake, or PA remains in the cell membrane and can protect from damage; thus, explaining PA’s significant protection from micronuclei formation and cell survival after gamma-rays.

Due to high density of ionization, high-LET carbon-ion radiation produces complex types of DNA damage and results in higher relative biological effectiveness. Carbon-ion radiation does not have a high fraction of indirect effects, but radical scavengers, DMSO and glycerol, could protect cells from cell death with low-LET gamma-irradiation (Fig. 5). These results may suggest that high-LET radiation can be protected by high concentration of radical scavengers by reducing the indirect effect. It is also possible that long-lived free radicals may contribute to DNA damage^27,28^. Since PA is lipophilic, the protection against carbon-ion radiation observed by PA can be explained by scavenging these long-live free radicals.

In conclusion, radical scavenging capacity is well related to DNA damage. GG and AA derivatives can reduce radiation-induced damage and cell death for gamma-rays. Among modified chemicals, only PA significantly reduced cell death induced by high-LET carbon-ion radiation. Further studies are required to verify the mechanisms of radioprotection by palmitoyl ascorbic acid 2-glucoside for high-LET carbon-ion radiation.

## Material and Methods

### Chemicals

DMSO was purchased from Fisher Scientific Co. (Fair Lawn, NJ). Glycerol was purchased from Mallinckrodt Specialty Chemicals Co. (St. Louis, MO). Glyceryl glucoside, ascorbic acid and its derivatives were synthesized and obtained by Tokyo Sugar Co., Ltd. (Tokyo, Japan) and Carlit Holding Co., Ltd. (Tokyo, Japan). Chemical structures were illustrated in Fig. 1. Concentrations on chemicals are weight per volume percentages. *In vitro* DNA experiment requires more dilute concentrations on chemicals than cell culture experiments. This is due to the ease chemicals have when entering the cell membrane. Higher concentrations of chemicals will lead to cytotoxic effects. Stock solution was prepared in 1% solution such as AA (56.7 mM), AA2G (30 mM), GA (40 mM), and PA (17 mM). Lambda DNA was purchased from Nippon Gene Co., Ltd. (Tokyo, Japan).

### Irradiation

Gamma-ray irradiation was performed at Colorado State University (Fort Collins, CO) with a J.L. Shepherd Model Mark I-68 nominal 6000 Ci ^137^Cs irradiator (J.L. Shepherd and Associates, San Fernando, CA) and used at room temperature (20 °C)^14,29^. The dosage rate was 2.5 Gy/min for cell survival and micronuclei experiments and 12.5 Gy/min for gel electrophoresis experiments. At the National Institute of Radiological Sciences (NIRS) in Chiba, Japan, particle-based irradiation experiments were carried out. For high-LET carbon-ion exposure, the Heavy-Ion Medical Accelerator in Chiba (HIMAC; Chiba, Japan) irradiated accelerated carbon ions at room temperature (20 °C)^29^. Specifics in regards to the beam characteristics of the particle radiation, biological irradiation procedures, and dosimetry have been depicted previously^30–32^. Carbon ions were accelerated at 290 MeV/nucleon of initial energy and spread out with a ridge filter for 6 cm width of SOBP^29^. The monolayer cell culture was irradiated at the center (50 keV/μm of average LET) within the SOBP at a distance of 119 mm from the entrance^33^. Dose rates for high-LET carbon-ions was set at 1 and 5 Gy/min respectively.

### Cell culture

Chinese hamster ovary (CHO), CHO10B2 (wild type) were provided by Dr. Joel Bedford at Colorado State University (Fort Collins, CO). CHO cells were maintained in culture in α-minimum essential medium (Gibco, Grand Island, NY), and supplemented with 10% heat inactivated fetal bovine serum (Sigma, St. Louis, MO) and supplemented with 100 U/ml penicillin, 100 µg/ml streptomycin and 25 ng/ml Amphotericin B at 37 °C and 5% CO_2_. The CHO cell doubling time is approximately 12 h.

### Electrophoresis and DNA analysis

One percent agarose gel with ethidium bromide and 1X TAE buffer was used. Freshly prepared for each experiment, 10 μl of the DNA solution consisted of 23 ng Lambda DNA (stock concentration 460 ng/μl), and 10 mM Tris-HCl buffer with DMSO, glycerol, GG, AA, 2G, GA, or PA. Each sample was exposed to gamma-rays, and run through electrophoresis after adding 2 μl of 6X DNA loading dye (15% Ficoll (w/v), 10% glycerol (v/v), 0.25% bromophenol blue (w/v), and 0.25% xylene cyanol FF (w/v) in distilled water). Electrophoresis was carried out at 100 V for 60 minutes in 1X TAE buffer. After electrophoresis, the gel was immersed with distilled water and placed in a 4 °C cold room for a maximum of 24 hours. After the cold room, the gel images were obtained with the Molecular Imager Gel Doc XR system with Image Lab software (Bio-Rad Laboratories, Inc., Hercules, CA). Images were analyses by pixel intensities. The amount of remaining intact DNA was calculated by dividing the treated samples by the control.

### Cell survival assay

Clonogenic assay was used to measure cell survival. To investigate the radioprotective cell survival after irradiation, CHO cells were pre-treated with DMSO, glycerol, GG, AA, 2G, GA, or PA for 0.5 h and irradiated with gamma-rays or high-LET carbon-ions. Cells were replated at a density devised to yield about 100 viable colony–forming cells/P–60 cell culture dish^34^. Seven to eight days after treatment, colonies were scored. To fix colonies, dishes were treated with 100% ethanol and stained with 0.1% crystal violet solution. Colonies encompassing greater than 50 cells were recorded as non-toxic reproductively viable surviving cells^34^. Cell survival fraction was obtained by dividing the irradiated samples by the control. Cell survival curves were produced using linear quadratic regression equations with Prism 6 (GraphPad Software, Inc., La Jolla, CA). The dose to achieve 10% survival fractions (D_10_ values) were obtained by interpolation of cell survival. Protection Enhancement Ratio (PER) values were calculated by D_10,control_ divided by D_10,drug treatment_.

### Micronuclei assay

To investigate the induction of micronuclei by irradiation, CHO cells were pre-treated with DMSO, glycerol, GG, AA, 2G, GA, or PA for 0.5 h and irradiated with gamma-rays or high-LET carbon-ions. Cells were treated with 4 μg/ml of Cytochalasin B (Sigma, St. Louis, MO) for 22 h^16^. Cells were then harvested in 5 ml of 75 mM KCl solution, centrifuged at 1,000 rpm for 5 min, and then fixed in 3:1 methanol: acetic acid solution and formaldehyde (Fisher Scientific, Fair Lawn, NJ)^16^. Next, cells were dropped onto slides and air dried at room temperature. Slides were stained in filtered 5% Giemsa solution in GURR solution (Gibco, St. Louis, MO) for 5 min^16^. Three hundred binucleated cells were scored per treatment dosage using a Zeiss Axioskop microscope. Images were taken by SPOT CCD camera RT 2.3.1 with SPOT basic software (Diagnostic Instruments. Sterling Heights, MI).

### Statistical analysis

All experiments were carried out more than three times, independently. Statistical comparison of mean values was performed using a one-way analysis of variance (ANOVA) with GraphPad Prism 6 (GraphPad Software, Inc., La Jolla, CA). P < 0.05 was considered to indicate a statistically significant difference. Error bars indicate the standard error of the mean.

## Electronic supplementary material

Supplementary Information

## Data Availability

All data generated or analyzed during this study are included in this published article.
