# Supplementary material for: Palmitoyl ascorbic acid 2-glucoside has the potential to protect mammalian cells from high-LET carbon-ion radiation
Source: Sci Rep. 2018 Sep 14;8:13822. doi: 10.1038/s41598-018-31747-1 (PMC6138748; doi:10.1038/s41598-018-31747-1)
Supplement: Supplementary file 1 — Supplementary Information [file 41598_2018_31747_MOESM1_ESM.docx]

Supplementary information for

Title: Palmitoyl ascorbic acid 2-glucoside has the potential to protect mammalian cells from high-LET carbon-ion radiation.

Alexis H. Haskins^1^, Dylan J. Buglewicz^1^, Hirokazu Hirakawa^2^, Akira Fujimori^2^, Yasushi Aizawa^3^, Takamitsu A. Kato^1^,

1. Department of Environmental & Radiological Health Sciences, Colorado State University, 1618 Campus Delivery, Fort Collins, CO, 80523, USA.

2. National Institute of Radiological Sciences, National Institutes for Quantum and Radiological Science and Technology, Chiba, 263-8555, Japan

3. Department of Planning & Development, Carlit Holdings Co. Ltd., 1-17-10 Kyobashi, Chuo-ku, Tokyo 104-0031, Japan

Corresponding Author:

Takamitsu A Kato, Ph.D.

Associate Professor

Department of Environmental & Radiological Health Sciences

Colorado State University

1618 Campus Delivery, Fort Collins CO 80523 USA

970-491-1881, [Takamitsu.Kato@Colostate.edu](mailto:Takamitsu.Kato@Colostate.edu)

**Supplementary Figure S1**: Full length gel images of Fig 1ABC. Red rectangle shows the cropping location.
